# Substantial understory contribution to the C sink of a European temperate mountain forest landscape

**DOI:** 10.1007/s10980-019-00960-2

**Published:** 2020-02-03

**Authors:** T. Dirnböck, D. Kraus, R. Grote, S. Klatt, J. Kobler, A. Schindlbacher, R. Seidl, D. Thom, R. Kiese

**Affiliations:** 1 Department for Ecosystem Research and Environmental Information Management, Environment Agency Austria, Spittelauer Lände 5, 1090 Vienna, Austria; 2 Karlsruhe Institute of Technology, Institute of Meteorology and Climate Research, Atmospheric Environmental Research (IMK-IFU), Kreuzeckbahnstraße 19, 82467 Garmisch-Partenkirchen, Germany; 3 Department of Forest Ecology, Federal Research and Training Centre for Forests, Natural Hazards and Landscape (BFW), Seckendorff-Gudent Weg 8, 1131 Vienna, Austria; 4 Department of Forest- and Soil Sciences, Institute of Silviculture, University of Natural Resources and Life Sciences (BOKU) Vienna, Peter-Jordan Straße 82, 1190 Vienna, Austria; 5 Rubenstein School of Environment and Natural Resources, University of Vermont, 81 Carrigan Drive, Burlington, VT 05405 USA; 6 Ecosystem Dynamics and Forest Management Group, School of Life Sciences, Technical University of Munich, Hans-Carl-von-Carlowitz-Platz 2, 85354 Freising, Germany

**Keywords:** Net ecosystem production, Carbon sequestration, Mountain forest, Herb layer, Tree regeneration, Forest disturbance, Ecosystem modelling

## Abstract

**Context:**

The contribution of forest understory to the temperate forest carbon sink is not well known, increasing the uncertainty in C cycling feedbacks on global climate as estimated by Earth System Models.

**Objectives:**

We aimed at quantifying the effect of woody and non-woody understory vegetation on net ecosystem production (NEP) for a forested area of 158 km^2^ in the European Alps.

**Methods:**

We simulated C dynamics for the period 2000–2014, characterized by above-average temperatures, windstorms and a subsequent bark beetle outbreak for the area, using the regional ecosystem model LandscapeDNDC.

**Results:**

In the entire study area, woody and non-woody understory vegetation caused between 16 and 37% higher regional NEP as compared to a bare soil scenario over the 15-year period. The mean annual contribution of the understory to NEP was in the same order of magnitude as the average annual European (EU-25) forest C sink. After wind and bark beetle disturbances, the understory effect was more pronounced, leading to an increase in NEP between 35 and 67% compared to simulations not taking into account these components.

**Conclusions:**

Our findings strongly support the importance of processes related to the understory in the context of the climate change mitigation potential of temperate forest ecosystems. The expected increases in stand replacing disturbances due to climate change call for a better representation of understory vegetation dynamics and its effect on the ecosystem C balance in regional assessments and Earth System Models.

**Electronic supplementary material:**

The online version of this article (10.1007/s10980-019-00960-2) contains supplementary material, which is available to authorized users.

## Introduction

Earth System Models (ESM) substantially improved our understanding of ecosystem carbon (C) cycling feedbacks on global climate (Flato et al. 2013; Bonan and Doney 2018). Nevertheless, many of the biogeophysical feedbacks remain to be addressed in a better way (Steffen et al. 2018). In forests, among the biggest challenges is the identification of C dynamics related to the understory including shrubs, herbs, grasses and small trees (Thrippleton et al. 2016; Landuyt et al. 2018). Forest ground vegetation contributes to ecosystem production and litter input, mediating carbon and nutrient dynamics (Nilsson and Wardle 2005; Gilliam 2007). At the same time, tree regeneration in the forest understory and ground vegetation are crucial for swiftly recovering C stocks after stand replacing forest disturbance (Edburg et al. 2012). However, the magnitude of uncertainty in modelled net ecosystem production (NEP) estimates as a result of disregarded processes mediated by forest understory is still unknown.

Forest stand replacing disturbances are classical examples where the understory determines C and nutrient dynamics because trees in the mid- and understory can take advantage of elevated light, water and nutrient availability, rapidly increasing photosynthetic activity and growth (Brown et al. 2010; Edburg et al. 2011; Mathys et al. 2013; Williams et al. 2014). However, a number of factors can limit tree regeneration, thereby causing a delay in the recovery of the C sink strength (Mayer et al. 2014; Matthews et al. 2017). Tree regeneration in temperate forests is often limited by browsing of large ungulates (Ammer 1996; Motta 1996; Friedrich Reimoser and Gossow 1996), seed predation by small mammals (Nopp-Mayr et al. 2012), or a scarcity of microsites suitable for germination (Diaci et al. 2005; Kupferschmid and Bugmann 2005). In addition, understory grasses and herbs (ground vegetation) can thwart tree regeneration after disturbance through competition (Ammer 1996; Pröll et al. 2015; Reimoser and Gossow 1996; Turner et al. 1997), with potential negative effects on NEP. At the same time, ground vegetation contributes to forest C sequestration (Nilsson and Wardle 2005; Gilliam 2007). The effect of ground vegetation on C sequestration also increases after tree replacing disturbances. This has been shown for lodgepole pine forests after a mountain pine beetle outbreak in British Colombia, Canada (Bowler et al. 2012), for a clearcut in North-Eastern US mixed conifer-hardwood forest (Williams et al. 2014), for wind throw areas in the High Tatra Mountains, Slovakia (Don et al. 2012), and for disturbed Norway spruce forests of the Kalkalpen National Park, Austria (Zehetgruber et al. 2017).

The above-cited studies substantially improved our knowledge about understory effects on C dynamics. However, all of them were plot-scale studies, and many were based on eddy flux measurements typically located in topographically flat and homogenous areas (Edburg et al. 2012). The effects of forest understory on NEP can therefore not easily be generalized over large, complex landscapes characterized by variation in site conditions, stand age, disturbance impact, understory plant functional attributes, and the ability of tree species to regenerate (Edburg et al. 2012; Williams et al. 2014). Moreover, disturbances are likely to gain importance as drivers of understory processes affecting NEP in temperate forests of Europe since wind events (Gregow et al. 2017) and bark beetle outbreaks are predicted to increase under climate change (Seidl and Rammer 2017; Seidl et al. 2014).

This paper presents, to our knowledge, the first landscape-scale study quantifying the effects of tree regeneration and ground vegetation development on temperate forest NEP using the ecosystem model system LandscapeDNDC (Grote et al. 2009; Haas et al. 2013) that includes process-based submodels for the simulation of forest carbon, nitrogen, and water cycles. Various sources of information such as airborne images, long-term field and LiDAR data were used to initialize and calibrate the model. We applied LandscapeDNDC on an area of 158 km^2^ at Kalkalpen National Park, Austria, a complex mountain region where disturbances have altered forest structure and function in the recent past and are expected to increase in the coming decades driven by climate change (Thom et al. 2017b). We focused our analysis on a 15 year time period (2000 to 2014), characterized by the three warmest years (2005, 2010, 2014) in 165-year global instrumental records (WMO 2015), an extreme heat wave in 2003 (Ciais et al. 2005), and high disturbance activity.

We hypothesized that forest understory increased the regional NEP through higher net primary production (NPP) and C input to the soil. We expected a higher NEP of the landscape after disturbance by including both, tree regeneration (Brown et al. 2010; Edburg et al. 2011; Mathys et al. 2013; Williams et al. 2014) as well as growth of ground vegetation (Bowler et al. 2012; Don et al. 2012; Williams et al. 2014; Zehetgruber et al. 2017) in the simulations. We also hypothesized that understory growth of trees and ground vegetation do not result in simple additive effects on NEP but that ground vegetation supresses C uptake of tree regeneration, particularly shortly after disturbance events (Ammer 1996; Reimoser and Gossow 1996; Pröll et al. 2015; Thrippleton et al. 2016).

## Materials and methods

### Study area

The Kalkalpen National Park is located at N47.47° E14.22° in the Northern Limestone Alps of Austria. The complex mountainous landscape, with elevations ranging from 385 to 1963 m a.s.l., is mostly forested (81%). Mean annual temperature ranges between 3.6 and 9.0 °C and annual precipitation between 1205 and 1741 mm (Thom et al. 2017b). Soils are predominantly shallow with Lithic and Rendzic Leptosols and Chromic Cambisols as the dominant soil types over carbonate bedrock. In our study, we focussed on all forested areas < 1200 m a.s.l. covering 158 km^2^ dominated by montane European beech (*Fagus sylvatica* (L.)) and mixed spruce (*Picea abies* (L. Karst.))—silver fir (*Abies alba* (Mill.))—beech forest types. The investigated time period between 2000 and 2014 included disturbance events triggered by the storms Kyrill, Paula and Emma, which hit Central Europe in the years 2007 and 2008. A subsequent bark beetle outbreak—fanned by the storm events—lasted from 2007 to 2012 (Seidl and Rammer 2017).

### LandscapeDNDC model description

To estimate growth of over- and understory trees as well as ground vegetation, and to distinguish between soil and plant respiration, we applied the ecosystem model system LandscapeDNDC (Grote et al. 2009; Haas et al. 2013). LandscapeDNDC has been used to determine forest development under undisturbed (Grote et al. 2011; Molina-Herrera et al. 2015) as well as disturbed conditions (Lindauer et al. 2014) and to estimate associated emissions of atmospheric trace gases (Kraus et al. 2015; Molina-Herrera et al. 2015) as well as leaching losses (Kiese et al. 2011; Dirnböck et al. 2016). Regional LandscapeDNDC applications are grid-based assuming that each simulated grid cell is an independent homogenous simulation unit representing a defined plant-soil system without lateral exchange of water, energy and matter. Within LandscapeDNDC, core models are MeTr^x^ (Kraus et al. 2015) and PSIM—Physiological Simulation Model (Grote 2007) describing soil biogeochemical and vegetation processes, respectively. The following paragraphs briefly describe the most important concepts and model adaptions regarding vegetation and hydrology that are relevant for this study.

#### Cohort approach

PSIM characterizes the vegetation in a grid cell in form of homogeneously distributed cohorts, i.e., groups of uniform morphology that represent different species or different dimensions (e.g. trees of different social classes) as well as ground covering species such as grasses and herbs (Grote et al. 2011). The vegetation within one simulation unit can thus be represented by multiple coexisting cohorts. The number of cohorts needs to be initialized and is constant during the simulation. This also means that cohorts are not merged even if they would develop into similar dimensions (for details see section model input). Biomass development of all cohorts (mature overstory trees, understory trees, and ground vegetation) is basically described by the same processes, which are photosynthesis and phenology, respiration, allocation and senescence (see Grote et al. 2009 and references therein), considering in principal the same plant organs (wood, foliage, fine roots, reserves, etc.).

#### Dimensional growth

For tree cohorts (over- and understory), structural growth (height, stem and crown diameter, etc.) is calculated for an average representative tree based on the biomass changes of the woody compartment using the allometric relationships presented by (Bossel 1996; Grote et al. 2011). The allocation into woody tissue is calculated based on the pipe-model theory (Shinozaki and Yoda 1964) that assumes a species-specific ratio between sapwood area and foliage and is driven by phenological development (Grote 2007). Because this ratio is set to zero for ground vegetation, no wood formation (and thus no woody biomass and no structural growth) is computed for grass and herbaceous ground vegetation cohorts.

For tree cohorts, area coverage directly results from crown dimensions and number of individuals. Due to missing structural growth of ground vegetation cohorts, an empirical function was developed that dynamically scales the area coverage of ground vegetation (A_h_) depending on overstory tree coverage (A_o_), using the findings of Helm et al. (2017). This function was based on 54 permanent forest plots (10 × 10 m), presenting a robust relationship of A_o_ and A_h_ for the study region (see details in S2).

#### Linking dimensional growth and foliage biomass

Dimensional growth of cohorts determines the upper limit of newly formed leaves from stored material at budburst. This cohort-specific value is given as a parameter for a closed canopy and restricted by crown volume for tree species cohorts and area coverage for ground vegetation cohorts.

#### Biomass distribution

Each cohort has its biomass distributed within the canopy space that is differentiated into layers of equal height. The distribution of leaf biomass over the length of the crown is modelled with a distribution function based on a species-specific parameter and crown length as a variable (Grote 2003, 2007). In case of ground vegetation, leaf biomass is completely allocated to the first canopy layer above ground due to missing structural growth. Leaf area per canopy layer is afterwards determined from biomass and specific leaf area. For trees, specific leaf area develops linearly from a minimum at the treetop to a maximum at crown base, while specific leaf area is set constant for grass and herbaceous ground vegetation.

#### Competition

Micrometeorological-, water- and nitrogen balance calculations determine climatic conditions and resource availability in each of these layers that affect—but are also influenced by—the cohort’s properties and interactions. Thus, all cohorts are in competition with each other. For example, light availability and thus photosynthesis in one canopy layer depends on the amount and properties of the foliage in higher layers. Belowground, soil water and nitrogen in a layer is only accessible for a plant cohort when sufficient fine roots are present.

#### Hydrology

In addition to model adaptations regarding area cover of ground vegetation, the tipping bucket approach of vertical soil water movement (Kiese et al. 2011) is replaced by a Van Genuchten approach to describe water percolation more realistically. The adaptation of the soil hydrology descriptions (details are provided in S1) was motivated by numerical problems of the tipping bucket approach for the simulation of soils with high stone contents, which are widespread in the study region.

### Simulation design

The study region was discretized by a regular 100 × 100 m grid resulting in a total of 15,793 simulation units. The vertical discretization of the soil and canopy domain was grid cell specific depending on vegetation and soil characteristics available from surveys. The height of the canopy domain is dynamically calculated depending on the maximum height of prevalent vegetation cohorts. A maximum of 40 equally sized layers is used for the canopy discretization. The vertical resolution of the soil domain depends on total soil depth but generally 0.5 cm layers were set for upper soil (O and A horizons) and 10 cm layer dimension for lower soil (B horizon). Hourly simulations spanned 15 years covering the time period 2000-2014. In order to explore the potential effect of forest ground vegetation and tree regeneration on ecosystem NEP, four hypothetical scenarios were set up (Table 1).

**Table 1:** Model scenarios including (+) or excluding (−) forest ground vegetation (i.e. herbs and grasses) and/or tree regeneration

Scenario name	Ground vegetation	Tree regeneration^a^
HR^a^	+	+
H	+	−
R^a^	−	+
NN	−	−

^a^Note that in addition to the four scenarios, tree regeneration was initialized assuming different densities (500 to 3000 trees ha^−1^), thereafter indicated as R_500_, HR_500_, R_1000_, HR_1000_, etc.

### Model input

#### Vegetation

The initialization of the vegetation was based on Thom et al. (2017b), who compiled a wall-to-wall estimate of vegetation structure and composition from forest inventory and planning data, aerial image analysis, and LiDAR data with a spatial resolution of 10 × 10 m. We aggregated this data to a spatial resolution of 100 × 100 m (Table 2; see details in S2). The maximum number of simulated vegetation cohorts per grid cell was set to seven, one cohort representing ground vegetation (only for the HR and H scenario), two cohorts representing tree regeneration (only for the HR and R scenario), and four cohorts representing overstory trees (see details in S2). The overstory cohorts represent the two most dominant tree species (depending on aboveground biomass shares) in the two most dominant height classes per grid cell. In the HR and R scenario, two tree regeneration cohorts (tree saplings of the type of the respective overstory) were included in all grid cells at the beginning of the simulation. Since LandscapeDNDC doesn’t provide a dynamic regeneration module and also because much regeneration is carried out or supported by management, new trees had to be initialized specifically. A total of 2500 tree saplings (height = 0.5 m) were initialized because it is the recommended density for sustainable tree regeneration according to regional forestry guidelines (Jasser and Diwold 2014). We considered a proportional partitioning of tree species into the two regrowth cohorts according to the biomass of the respective tree species in the overstory. In reality, however, a lower density is often realized, so that we varied the tree sapling density across simulations from 500 to 3000 ha^−1^ (500, 1000, 1500, 2000, 2500, and 3000). The respective scenarios were indicated as R_500_, HR_500_, R_1000_, HR_1000_, etc. Note, that browsing damage to tree saplings by large ungulates was not modelled.

**Table 2:** Initial stem volume (vol), C pool quantities of aboveground stem wood (st), branch wood (br) + foliage biomass (fl), roots (C below), and soil organic carbon stocks (SOC)

	Overstory		Tree regeneration^a^		Ground vegetation	Total
vol (m^3^ ha^−1^)	225.7 ± 169.3		0.05 ± 0.02		–	225.7 ± 169.3
C above (t C ha^−1^)	st	55.3 ± 43.3	st	0.03 ± 0.01	0.25 ± 0.03	73.3 ± 58.6
	br + fl	17.6 ± 16.2	br + fl	0.05 ± 0.05		
C below (t C ha^−1^)	14.0 ± 10.3		0.03 ± 0.01		0.09 ± 0.01	13.8 ± 10.2
SOC (t C ha^−1^)	–		–		–	119.7 ± 34.3

Values correspond to the mean and standard deviation across the complete study region

^a^2500 tree individuals ha^−1^

Disturbance effects on vegetation were taken from a previous study carried out in the area (Thom et al. 2017b). This data contained spatially explicit yearly information on disturbed tree volume and disturbance type. The data resolution of 10 × 10 m was additively scaled to the 100 × 100 m grid size to fit LandscapeDNDC simulations (see details in S2).

#### Soils

LandscapeDNDC requires soil-depth-specific initial information of major soil properties, i.e., organic carbon and nitrogen contents, bulk density, pH, texture and soil hydrologic parameters. This information was derived based on soil map data from Kobler (2004), which was available on a spatial resolution of 100 × 100 m (see details in S2) (Table 2).

#### Weather and atmospheric properties

We used daily weather data from Thom et al. (2017b). Nitrogen deposition was represented by a mean nitrogen concentration in precipitation (2.87 mg l^−1^ N) taken from Dirnböck et al. (2016). Atmospheric concentration of CO_2_ was set constant to 370 ppm.

### Model parametrization and evaluation

Parametrization for spruces and ground vegetation has been documented in Lindauer et al. (2014). Additional parameters for beech have been obtained from various sources and evaluated e.g. in Grote et al. (2011). Calibration of few allometric parameters for the current study region has already been carried out in a previous study with LandscapeDNDC (Dirnböck et al. 2016). Some minor adjustments, such as the desired height:diameter ratio were set from site measurements to account for the differences between slope and plateau. For evaluation, we used several data sets with observations from the LTER Zöbelboden site (https://deims.org/8eda49e9-1f4e-4f3e-b58e-e0bb25dc32a6), which is a 90 ha long-term ecosystem research area in the Kalkalpen National Park. The local forest types are representative for the bulk of montane forests in the park (Jost et al. 2011; Kobler et al. 2015; Dirnböck et al. 2016; Zehetgruber et al. 2017). Data from two long-term monitoring plots were used for the calibration of tree growth (IP1: 1996-2010 and IP2: 1998-2016), and soil respiration (IP1: 2009-2011; IP2: 2015) (see details in S3). IP1 is located on a flat plateau (950 m a.s.l.) stocked by a 115-year-old spruce-beech forest and Chromic Cambisols and Hydromorphic Stagnosols as the main soil types. IP2 is located adjacent to IP1 on a 36° steep slope, dominated by an old growth, mixed beech-maple-ash-spruce forest on shallow Lithic and Rendzic Leptosols. Both plots experienced wind and bark beetle disturbances to varying degrees (Kobler et al. 2015). For the comparison of measured versus modelled tree growth, soil moisture, and soil respiration, we used the R package hydroGOF version 0.3-10 (Zambrano-Bigiarini 2017). Mean error, Pearson correlation coefficient and the Kling-Gupta efficiency (Gupta et al. 2009) were used as indicators to evaluate model performance. The King-Gupta efficiency is an aggregated measure expressing correlation, variability and bias concerning the comparison of modelled simulations with empirical data. Long-term 10 × 10 m (n = 54; 1993–2014) records of forest vegetation (Helm et al. 2017) were used for the function that scales the ground coverage of ground vegetation depending on overstory tree coverage. Vegetation records at IP1 and IP2 were used for validation of ground vegetation biomass dynamics (see details in S3).

### Data analyses

While the amount of C fixed by photosynthesis in an ecosystem is defined as gross primary production (GPP) and NPP results from GPP minus autotrophic respiration, we defined NEP as GPP minus total ecosystem respiration (the sum of autotrophic and heterotrophic respiration) according to Lovett et al. (2006). Cumulative regional NEP was calculated as the sum of all simulated grid cells over the period 2000 to 2014. Tree regeneration as well as ground vegetation effects on NEP were calculated by subtracting the respective scenarios from each other. For the calculation of understory effects after disturbance, we differentiated between undisturbed areas and areas with > 211 m^3^ ha^−1^ stem wood damage, representing 1% of the total study area, and overstory tree replacement for the average forest stand of the study area (Table 2).

## Results

### Model evaluation

Predicted tree stem biomass and soil respiration was well in accordance with observations at the two intensively studied plots IP1 and IP2 (S3). Stem biomass development between 1996 and 2010 (IP1) and between 1998 and 2016 (IP2) of the two dominant tree species could be modelled with a Pearson r > 0.99 and Kling-Gupta efficiency between 0.57 and 0.97. The model underestimated stem biomass (of *Picea abies* and *Fagus sylvatica*) with a mean error of 1% (IP1) and 33% (IP2) (Table 3). Note, that the degree of error in IP2 resulted from the difficulty to simulate the heterogeneous tree structure of the stand on the steep slope and does not reflect annual growth estimates in the same way, indicated by the high Pearson correlation coefficients. Daily soil respiration was modelled with a Pearson r = 0.97 and Kling-Gupta efficiency of 0.93 at IP1, whereas at IP2 Pearson r was 0.66 and Kling-Gupta efficiency was 0.36. At IP1, the model underestimated soil respiration with a mean error of 4.3% (IP1), and overestimated soil respiration with a mean error of 15.2% at IP2 (Table 3). Seasonal peak ground vegetation biomass as modelled with LandscapeDNDC corresponded well with measured biomass (S3) records at IP1 and IP2 showing deviations between 0.01 and 0.05 t ha^−1^, representing 1.3% and 8.3%, respectively (Table 4).

**Table 3:** Correspondence of modelled and measured stem biomass and soil respiration for the two intensive plots (IP1 and IP2)

	Stem biomass (kg m^−2^)		Soil respiration (kg C ha^−1^ day^−1^)
	PIAB	FASY	
IP1	1996–2010		2009–2011
Mean error	− 0.18	− 0.12	− 0.93
Pearson correlation coefficient	0.99	1	0.97
Kling-Gupta efficiency	0.97	0.89	0.93
IP2	1998–2016		2015
Mean error	− 0.24	− 3.22	4.58
Pearson correlation coefficient	0.99	0.99	0.66
Kling-Gupta efficiency	0.71	0.57	0.36

See S3 for details about observation data

PIAB: Norway spruce (*Picea abies* (L.) H. Karst.); FASY: European beech (*Fagus sylvatica* L.)

**Table 4:** Estimated and modelled summer season (June to August) ground vegetation biomass for the plateau (IP1) and slope plot (IP2)

Year	Ground vegetation biomass [t ha^−1^]	
	Estimated (mean)	Modelled (mean ± SD)
IP1		
2004	0.66	0.64 ± 0.10
2007	0.69	0.71 ± 0.11
2010	0.77	0.78 ± 0.12
IP2		
2004	0.60	0.55 ± 0.04

See S3 for the methods applied to estimate biomass

### Cumulated net ecosystem production (NEP)

**Fig. 1:**
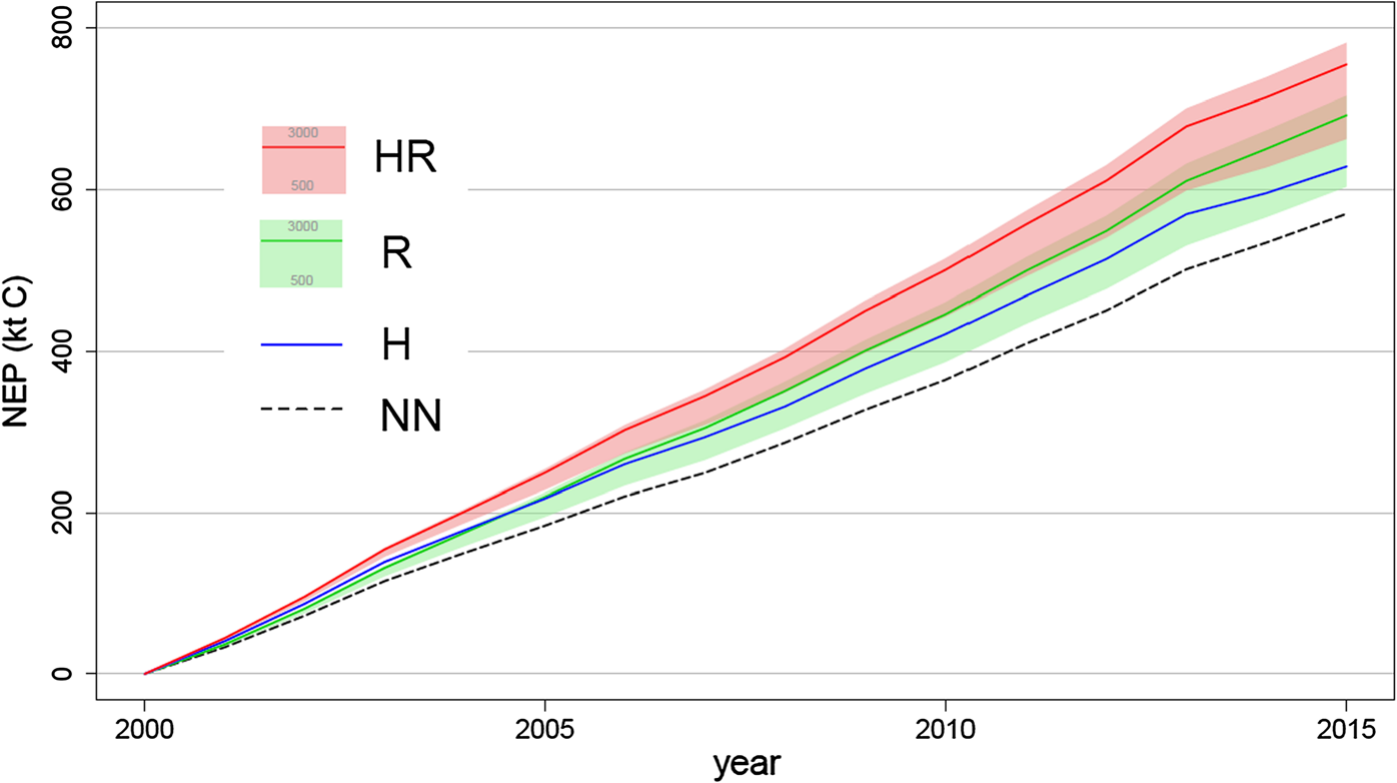
Cumulative net ecosystem production (NEP) of the study area for the four different scenarios. *NN* no ground vegetation or tree regeneration, *R* no ground vegetation but tree regeneration, *H* ground vegetation but no tree regeneration, *HR* ground vegetation and tree regeneration. Light green and light red shades show the R_500_ and R_3000_, and HR_500_ and HR_500_ scenario, respectively (subscripts indicate 500 and 3000 trees ha^−1^ regeneration). Solid lines represent 2500 trees ha^−1^ regeneration

**Fig. 2:**
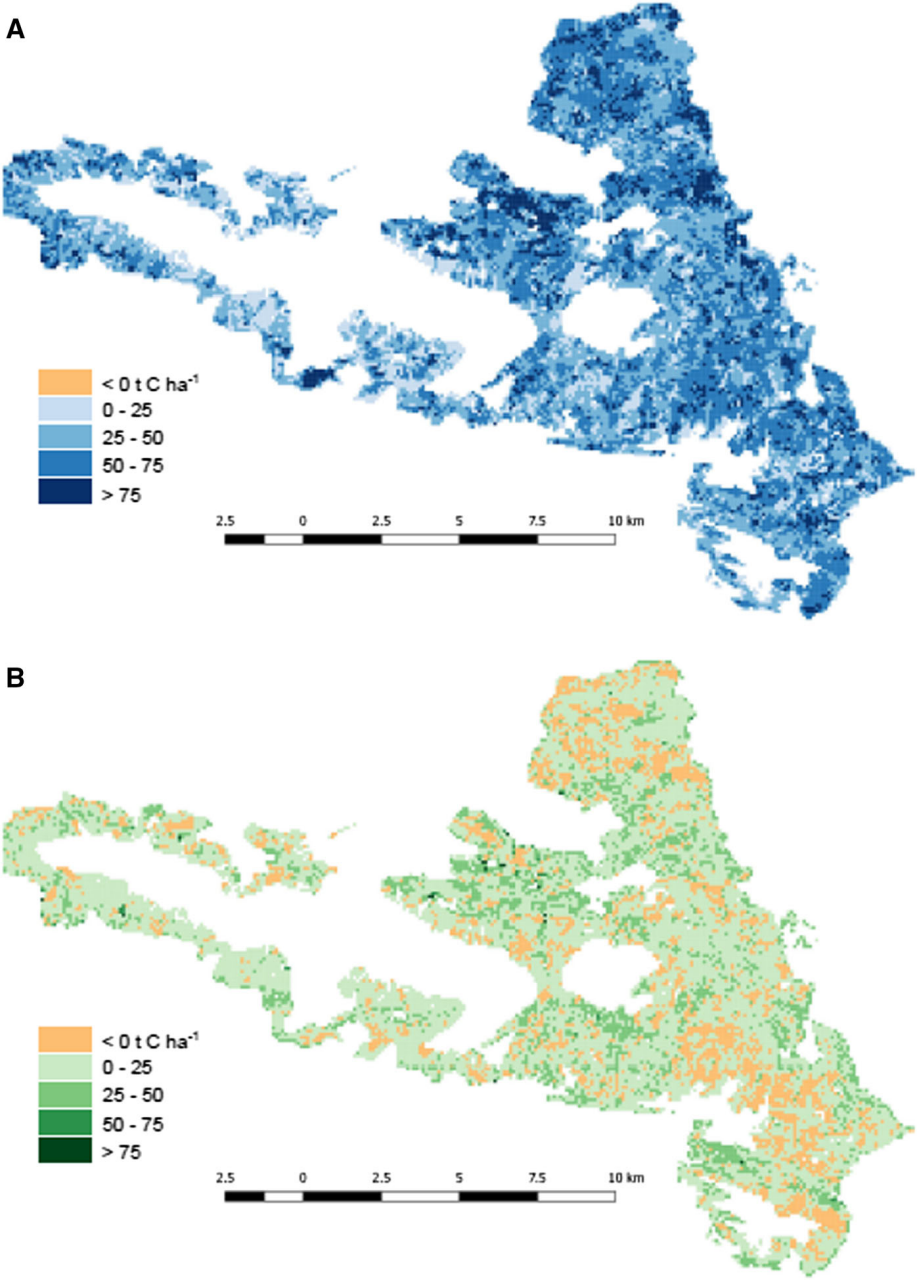
Cumulative net ecosystem production (NEP) from 2000 to 2014, **A** without tree regeneration and ground vegetation (NN scenario), and **B** effect of tree regeneration and ground vegetation on NEP. Positive values in **A** indicate net C sinks, negative values indicate net C sources (not visible due to its small extent). Positive values in **B** indicate higher NEP when accounting for tree regeneration and ground vegetation, negative values indicate lower NEP (in the range of 2.1 ± 1.7 t C ha^−1^). White area is outside the study region. Understory effect was calculated by HR_2500_–NN

In the HR_2500_ scenario, the mean C sink of the study region was 48.3 ± 20.8 t C ha^−1^ between 2000 and 2014 resulting in a total area cumulated NEP of 754 kt C (Figs. 1, 2a). The NN scenario resulted in a mean NEP of 36.5 ± 25.1 t C ha^−1^ adding up to a total of 570 kt C for the landscape (Fig. 1). Hence, NEP increased by 32% (11.8 ± 12.6 t C ha^−1^) when taking tree regeneration and the ground vegetation layer into account (Figs. 1, 2b). The sensitivity analysis with different tree regeneration densities resulted in a 16% (HR_500_) to 37% (HR_3000_) NEP increase (Fig. 1).

When accounting for tree regeneration without growth of ground vegetation, i.e. the R_2500_ scenario, mean NEP resulted in 44.3 ± 22.6 t C ha^−1^ between 2000 and 2014 summing to an area cumulated NEP of 692 kt C (Fig. 1). Hence, NEP increased by 21% when taking tree regeneration into account. The sensitivity analysis resulted in a 6% (R_500_) to 26% (R_3000_) NEP increase (Fig. 1).

When accounting for only ground vegetation, i.e. the H scenario, mean NEP resulted in a 40.3 ± 22 t C ha^−1^ sink for atmospheric carbon between 2000 and 2014 resulting in a cumulated NEP of 628 kt C of the landscape (Fig. 1). This represents a 10% higher NEP owing to ground vegetation.

The contribution of tree regeneration and ground vegetation to NEP varied across the landscape (Fig. 2b). On 25% of the area, the HR_2500_ scenario resulted in a decrease of the cumulated NEP compared to the NN scenario (mean of negative values: 2.1 ± 1.7 t C ha^−1^). In 3% of the area, the HR_2500_ scenario resulted in a decrease of the cumulated NEP compared to the H scenario (mean of negative values: 0.7 ± 1.2 t C ha^−1^).

**Fig. 3:**
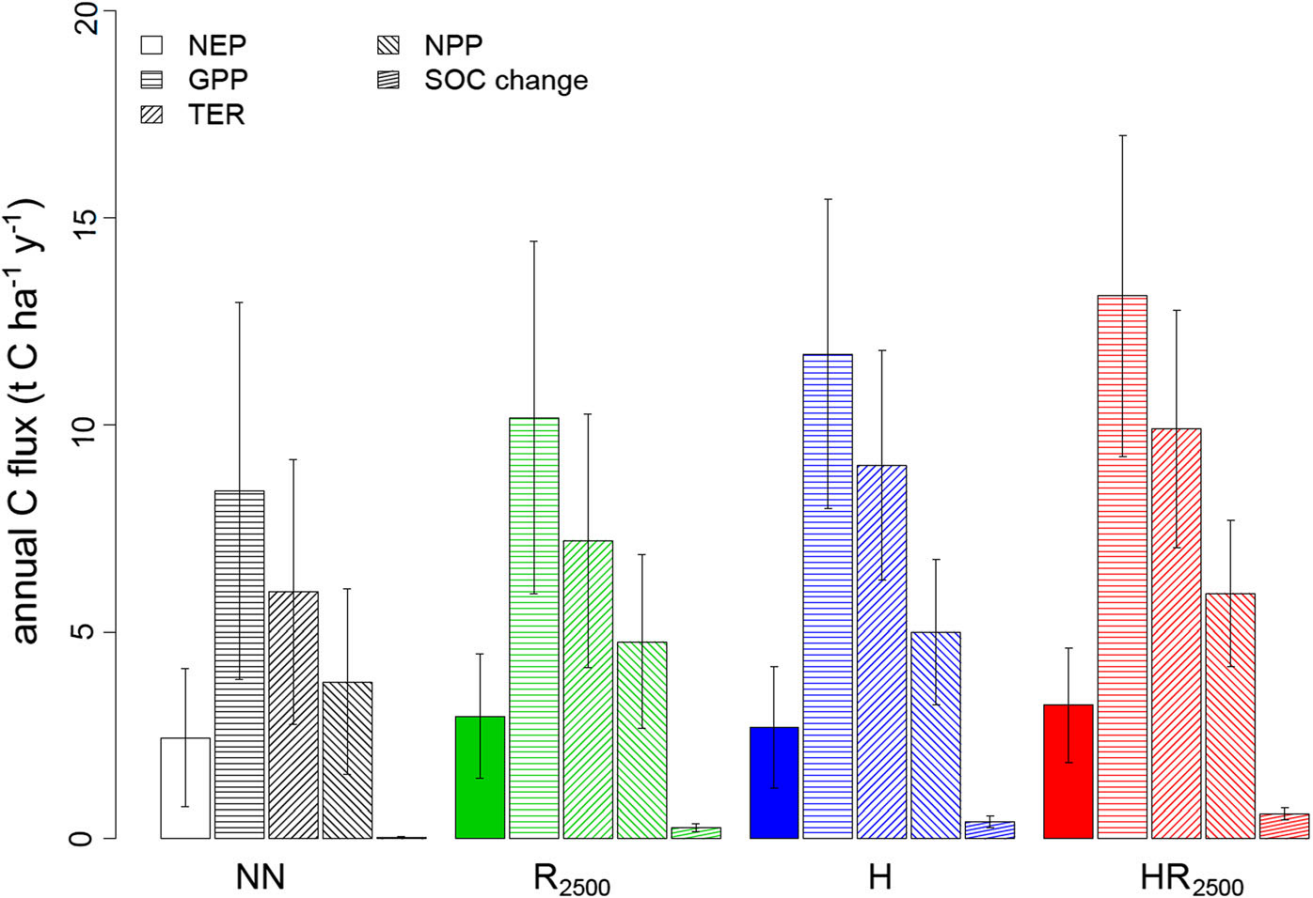
Magnitude (mean ± SD) of annual net ecosystem production (NEP), gross primary production (GPP), total ecosystem respiration (TER), net primary production (NPP), and changes in soil organic C (SOC) in the four scenarios. *NN* no herb layer or tree regeneration, *R*_*2500*_ no herb layer but tree regeneration, *H* herb layer but no tree regeneration, *HR*_*2500*_ herb layer and tree regeneration

Mean annual GPP, NPP, total ecosystem respiration (TER), and annual increments in soil organic C (SOC) increased in the order NN < R_2500_ < H < HR_2500_ scenario (Fig. 3). Mean annual NEP was lowest in the NN scenario (2.4 ± 1.7 t C ha^−1^ year^−1^) and highest in the HR_2500_ scenario (3.2 ± 1.4 t C ha^−1^y^−1^), but the R_2500_ scenario showed higher values (3.0 ± 1.5 t C ha^−1^ year^−1^) than the H scenario (2.7 ± 1.5 t C ha^−1^ year^−1^). The latter was due to high TER rates in the H scenario (Fig. 3) resulting in a lower mean NPP:GPP ratio for the H scenario (0.43) than the R_2500_ scenario (0.47). The contribution of SOC to NEP, indicated by ratios of annual SOC changes and NEP increased in the order NN (0.12) < HR_2500_ (0.14) < R_2500_ (0.18) < H (0.2). Mean annual NEP of the R_500_ to R_3000_ scenarios was lower than mean annual NEP of the H scenario only when less than 1000 tree saplings ha^−1^ were used.

### Understory effects on NEP after disturbance

**Fig. 4:**
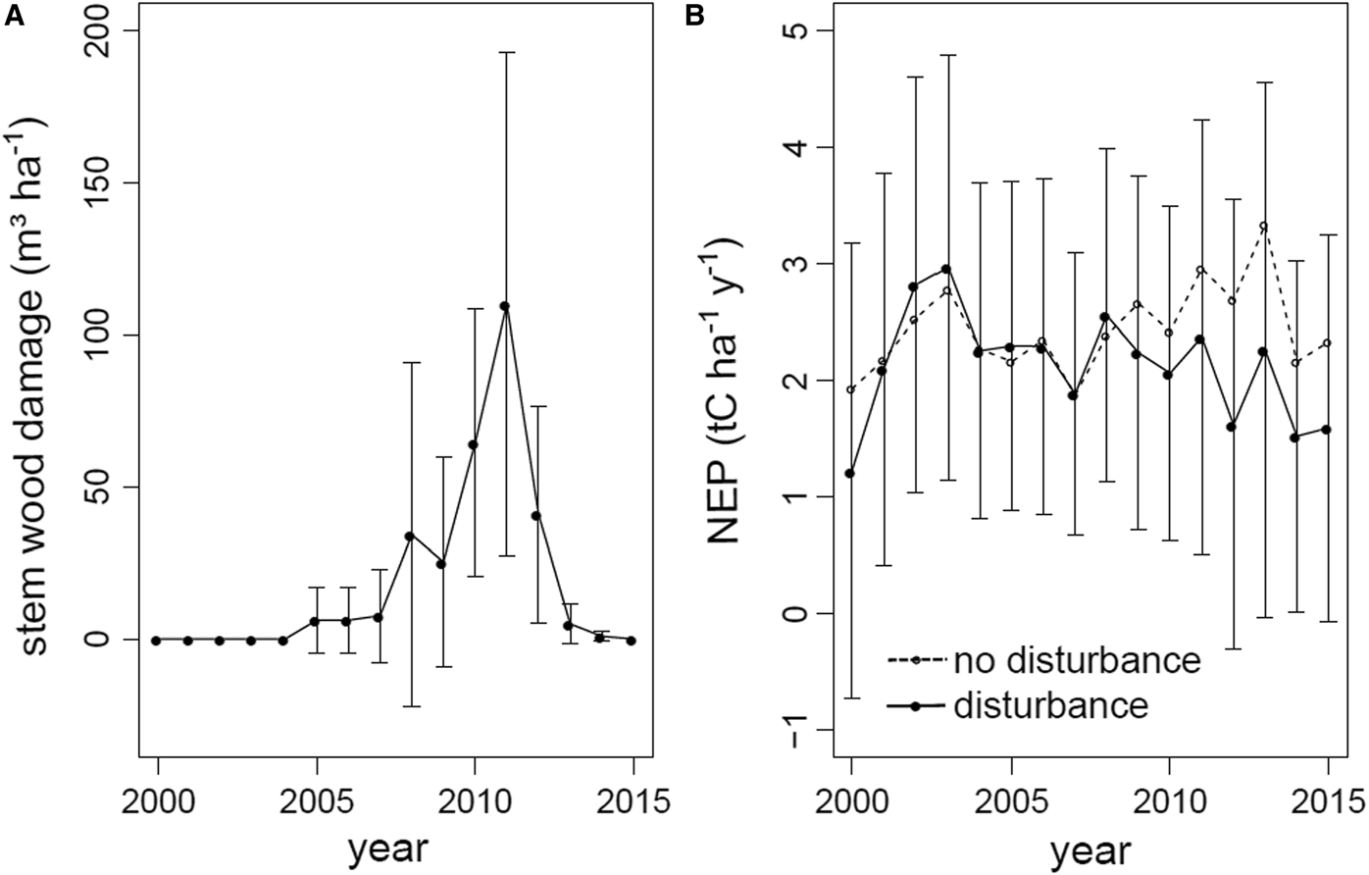
**A** Mean annual stem wood damage in disturbed areas of the Kalkalpen National Park (1% of the area with stem wood damage > 211 m^3^ ha^−1^ between 2005 and 2014) and **B** annual net ecosystem production (NEP) in disturbed (mean ± SD) and undisturbed (mean) areas in the NN scenario (i.e. without considering tree regeneration and ground vegetation)

In the NN scenario, i.e. without considering tree regeneration and ground vegetation, NEP was higher in undisturbed areas (2.49 ± 1.73 t C ha^−1^ year^−1^) than in disturbed areas (2.30 ± 1.67 t C ha^−1^ year^−1^) during the entire study period (2000 to 2014). With forest disturbance starting in the year 2005 and disturbance impact on growing stock peaking between 2010 and 2012 (Fig. 4a), NEP diverged more strongly between undisturbed and disturbed sites (Fig. 4b). After the year 2007, NEP in disturbed areas was on average 0.59 t C ha^−1^ year^−1^ lower than NEP in undisturbed areas (NN scenario).

**Fig. 5:**
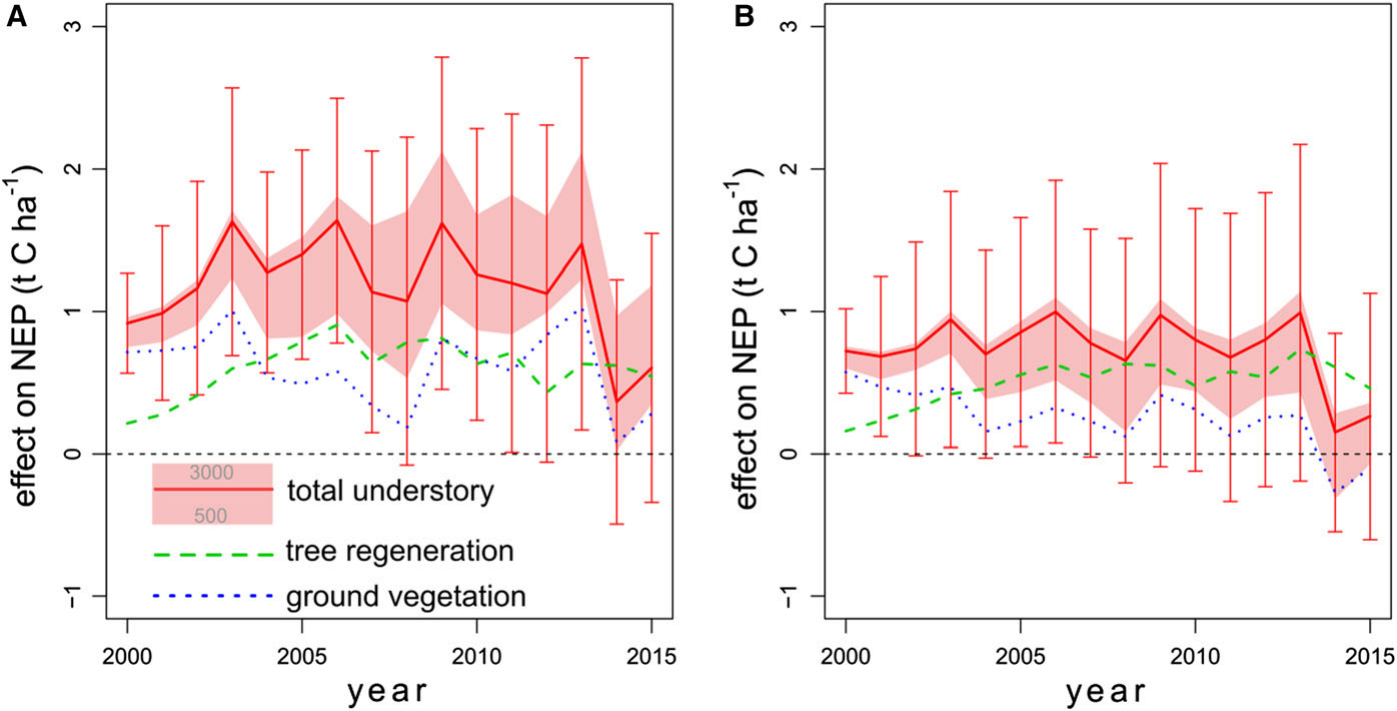
Regional mean combined (± SD) and single effects of tree regeneration and ground vegetation on annual net ecosystem production (NEP), under disturbance (**A** > 211 m^3^ ha^−1^ stem wood damage between 2005 and 2014) and without disturbance (**B**). Combined effects were calculated as the difference between scenario HR_500_ to HR_3000_ and NN (mean ± SD for HR_2500_–NN). Single effects of tree regeneration (green dashed line) were calculated as the difference between scenario HR_2500_ and H. Single effects of ground vegetation (blue dotted line) were calculated as the difference between scenario HR_2500_ and R_2500_

By evaluating joint effects of tree regeneration and ground vegetation at disturbed sites, we found that mean annual NEP was higher in the HR (HR_500_: 0.81 ± 0.31; HR_2500_: 1.18 ± 0.35; HR_3000_: 1.53 ± 0.37 t C ha^−1^ year^−1^) compared to the NN scenario (Fig. 5a). These effects were smaller in undisturbed sites (HR_500_: 0.37 ± 0.26; HR_2500_: 0.73 ± 0.24; HR_3000_: 0.82 ± 0.24 t C ha^−1^ year^−1^) (Fig. 5b). Compared to NN, the combined contributions of tree regeneration and ground vegetation caused a 35 and 67% higher mean annual NEP for HR_500_ and HR_3000_ in disturbed areas while only 15 to 33% for HR_500_ and HR_3000_ in undisturbed areas.

This difference was mainly driven by the accelerated growth of ground vegetation and annual litter input to the soil. Ground vegetation contributed 4.21 ± 1.13 and 2.7 ± 0.53 t C ha^−1^ year^−1^ to mean annual GPP in disturbed and undisturbed areas, respectively. In contrast, the difference in the contribution of the tree regeneration to mean annual GPP in disturbed and undisturbed areas was much lower (disturbed areas: 1.43 ± 0.47 t C ha^−1^ year^−1^; undisturbed areas: 1.31 ± 0.52 t C ha^−1^ year^−1^). Since the effect of forest ground vegetation on TER did not increase to the same extent, the NEP contribution of ground vegetation increased relative to tree regeneration in the disturbed forest area. Yet, the number of tree saplings had a strong positive effect on NEP after disturbances. It has to be noted that ground vegetation production was slightly higher in disturbed than in undisturbed areas already in the year 2000 due to a more open tree canopy (i.e. lower tree biomass causing higher ground vegetation coverage and biomass) in disturbed areas.

## Discussion

In our simulations, tree regeneration as well as ground vegetation increased the NEP through photosynthetic C uptake, particularly after canopy opening due to disturbance. This increase is in line with plot scale studies from other regions (Amiro et al. 2010; Edburg et al. 2011, 2012; Bowler et al. 2012; Don et al. 2012; Williams et al. 2014) as well as chronosequence measurements at Kalkalpen National Park (Zehetgruber et al. 2017). While these previous analyses focused on the plot to stand scale, we here showed that understory vegetation is of considerable relevance for the landscape-scale forest C balance. We achieved this by using mapped soil and vegetation data, a reconstruction of forest disturbances as drivers of the ecosystem model LandscapeDNDC, as well as by considering an environmentally driven development of overstory trees, newly established seedlings, and ground vegetation. Our results show that the cumulative NEP over 15 years was between 16% and 37% higher if accounting for the effect of tree regeneration and of ground vegetation compared to simulations without these components. The mean annual contribution of tree regeneration and ground vegetation to NEP (0.40 to 0.91 t C ha^−1^ year^−1^) was in the same order of magnitude as the average annual European (EU-25) forest C sink (0.75 t C ha^−1^ year^−1^) estimated by Luyssaert et al. (2010) for the period 1990 to 2005. Among the two understory components, tree regeneration contributed more strongly to NEP. However, in disturbed areas the effect of ground vegetation to NEP was in the same range. The landscape-scale C cycle contribution of trees versus ground vegetation was strongly determined by the number of tree saplings prior to disturbance as well as the density—and hence competitive strength—of ground vegetation.

### Understory effects on NEP

The modelled NEP under undisturbed forest conditions (between 2.4 ± 0.40 (NN scenario) and 3.2 ± 1.4 (HR_2500_ scenario) t C ha^−1^ year^−1^) was similar to rates typically found in field observations both in the study area (Kobler et al. 2015; Zehetgruber et al. 2017; Kobler et al. 2019) and in other mature temperate forest stands in Europe. As an example, Kowalski et al. (2004) reported a NEP of approx. 1 to 5 t C ha^−1^ year^−1^ in mature high forests in Britain, Finland, and France. Etzold et al. (2011) attributed 1.5 and 4.2 t C ha^−1^ year^−1^ of NEP to two mountain forests in Switzerland.

While disturbances due to wind and bark beetle began affecting the study area already in the year 2005, substantial loss of growing stock occurred only after 2007 (Fig. 4). Accordingly, annual NEP decreased by 0.7 ± 0.27 t C ha^−1^ year^−1^ after 2007 (NN scenario). The lowest NEP (− 4.0 t C ha^−1^ year^−1^ in the H and R_2500_ scenarios) occurred during and after the years with peak disturbance (years 2011 and 2012). This finding corresponds well with observations from others, who accounted also for ground vegetation by either using eddy covariance measurements or empirical modelling. Zehetgruber et al. (2017), studying a disturbance chronosequence within our study area, showed that a Norway spruce forest on deep Cambisols became a source of − 5.5 t C ha^−1^ year^−1^ 3 years after stand replacing disturbance. Furthermore, Matthews et al. (2017) observed a seasonal (May to October) NEP of − 4.1 and − 1.8 t C ha^−1^ year^−1^ three and four years after stand replacing wind throw, respectively. Comparable NEP rates (− 3.5 t C ha^−1^ year^−1^) were observed at a forest site in Germany two years after windthrow from the storm Kyrill (Lindauer et al. 2014), and after clear cutting in four European forests (− 4.3 to − 1.1 t C ha^−1^ year^−1^, Kowalski et al. (2004)). However, also higher levels of post-disturbance C loss have been reported, e.g. in a Swedish wind throw area for which Lindroth et al. (2009) estimated a NEP of up to − 10.8 t C ha^−1^ year^−1^.

While understory had a positive effect on NEP in most parts of our study area, the magnitude of the effect differed across the landscape and was higher in forests disturbed by wind or bark beetle than in undisturbed forests. Under the NN scenario, i.e. in the absence of tree saplings and ground vegetation, the recovery of C uptake after disturbance was largely a function of disturbance severity and site productivity, corroborating current knowledge (Anderegg et al. 2016). Also in line with other studies, understory trees surviving a disturbance event or establishing after disturbance lead to a steady recovery of the forest, eventually returning from a C source to a C sink (Amiro et al. 2010; Edburg et al. 2012; Williams et al. 2014; Dobor et al. 2018). Resulting from the elevated light, water and nutrient availability after disturbance, an increased growth of ground vegetation, and litter input to the soil, also plays a significant role in this recovery process. Comparable to previous studies (Bowler et al. 2012; Don et al. 2012; Williams et al. 2014; Zehetgruber et al. 2017), we found 35% (HR_500_) to 67% (HR_3000_) higher NEP in disturbed areas in contrast to 15 (HR_500_) to (HR_3000_) 33% in undisturbed areas, when considering both tree regeneration and ground vegetation. Notwithstanding the significance of the contribution of the forest understory to NEP in disturbed forests, high severity disturbances were restricted to a relatively small portion of the area in our study region (Thom et al. 2017a). Consequently, interactions between understory dynamics and natural disturbances did not substantially affect the landscape scale C sink strength in our study area yet having a large potential in the future (Seidl et al. 2014).

Non-woody plants exert competitive effects on tree regeneration (Pröll et al. 2015; Thrippleton et al. 2016), potentially precluding tree establishment after disturbance as shown in e.g. the Yellowstone National Park (Turner et al. 1997). In temperate mountain forest landscapes, these interactions are particularly important because open tree canopies frequently result in a dense herb and grass layer, rapidly increasing production following canopy opening (Ammer 1996). Usually, larger gaps result in higher understory biomass because of higher light availability, more growing space and less root competition (Collins and Pickett 1987; Ritter et al. 2005). In our simulations we have considered overstory regulation on understory growth as well as competition for water and nutrients between understory components. Since herbs and grasses have a higher relative share of active compartments (foliage and fine roots) than trees (that also consist of less respiring wood), respiration per unit biomass and specific turnover rates are higher. Thus, mean NPP:GPP ratios are smaller under scenarios that include ground vegetation (H and HR). This is in accordance with typical findings from temperate forests (Gilliam 2007). Consequently, the smallest NEP was obtained when only forest ground vegetation but no tree regeneration was considered (H scenario). This is in spite of high relative increases in SOC owing to the annual litter inputs (highest SOC:NEP ratios were found in the H scenario) which improves nutrient supply and has been observed to increase carbon assimilation efficiency (Vicca et al. 2012). However, nutrient demand of ground vegetation is also higher so that the overall nutritional state was not significantly changed. Ground vegetation could expand coverage rapidly after overstory disturbance resulting in a more positive immediate effect on GPP than on that of tree regeneration. However, the effect was compensated quickly from the increasing growth of the more efficient tree regeneration that also decreased growth of grass and herbs after a few years in the HR scenarios. Overall, the contributions of tree regeneration and ground vegetation to NEP were similar for the observed period. Considering the whole investigated area, the relatively small portion of severely disturbed areas didn’t decrease NEP significantly on a landscape scale, although the effect was considerable looking on disturbed areas alone. Hence, we hypothesize that the balance of positive and negative effects of the forest understory on NEP at the landscape scale will vary with region, disturbance regime as well as the abundance of natural tree regeneration. For temperate mountain forests it seems to be generally important to consider forest ground vegetation in addition to tree regeneration because they are often characterized by open tree canopies resulting in high levels of biomass in non-woody plants (Thrippleton et al. 2016).

### Limitations and potential model improvements

Based on our results showing that forest understory and their interaction substantially affects the forest C sink, we argue to consider related process also in Earth System Models, corroborating the conclusions in the review of Landuyt et al. (2018). Since forest disturbances disproportionately elevated the contribution of understory to NEP, and forest disturbances are expected to increase in the future due to climate change (Seidl et al. 2014), the consideration of understory processes in models becomes even more important. We here provided an example for a possible implementation in a process-based ecosystem model, but shortcomings as to the representation of certain processes still exist so that an extrapolation to other systems remains to be tested. First, since we compared vital tree regeneration with a hypothetical bare soil scenario, the magnitude of understory effects on NEP simulated here represents an upper bound estimate. However, even though the growth and survival of tree saplings can be limited by various factors, it is very unlikely that trees would fail to regenerate across our entire study area. By studying stem densities between 500 and 3000 saplings per hectare, we quantified the sensitivity of the NEP effect of various regeneration densities, representing realistic ranges. Nevertheless, future studies should incorporate further mechanisms limiting tree regeneration beyond light, water and nutrient availability (e.g. browsing, seed availability), as it has been considered in certain models of forest dynamics (Bugmann 2001; Seidl et al. 2012). LandscapeDNDC doesn’t provide a mechanistically driven regeneration model that would consider the appearance of new trees over longer periods and possibly include more species than could be considered in the current approach. This would allow for a more mechanistic representation of vegetation dynamics at the landscape scale (Thrippleton et al. 2016).

Second, we acknowledge that some uncertainty exists regarding the selection of cohorts, since it has been shown that a high aggregation might produce biased results under specific conditions (Wutzler 2008). However, more detail in stand structure increases the dependence on initialization and parameter accuracy and decreases generality of the model results. Therefore, the use of 2–3 cohorts in the few process-oriented ecosystem models that are available is still a common choice (Deckmyn et al. 2008; Jiang et al. 2019).

Third, we have not addressed differences in non-woody understory dynamics due to different species compositions between forest types. However, Seebacher et al. (2012) showed that varying water and nutrient conditions favour different plant functional types in our study area. It is very likely that the responses of these functional types to environmental perturbations and thus their impact on NEP will differ, likely resulting in variation in their effects on NEP. Exploiting differences in life history strategies when implementing understory dynamics in models is hence an important future direction of research (Landuyt et al. 2018). Third, the total amount of C loss to the atmosphere, apart from being determined by C uptake in plants, is also driven by altered decomposition dynamics of soil organic matter (Köster et al. 2011; Don et al. 2012). Disturbance can result in warmer and wetter soil conditions, enhancing the decomposition of SOM and thus soil respiration (Morehouse et al. 2008; Mayer et al. 2014). These effects can, however, not be broadly extrapolated because partial disturbances are common (Senf et al. 2018), causing only moderate changes in the microclimate and soil respiration (Kobler et al. 2015). Hence, disturbance severity and patch size play an important role for soil respiration in post-disturbance temperate forests (Sommerfeld et al. 2018). Since LandscapeDNDC is not fully able to reflect this heterogeneity in the disturbance regime of temperate forests (Sommerfeld et al. 2018) and instead simulates disturbance effects as a lowered LAI and a homogeneously increased gap fraction, an underestimation of soil warming and thus soil respiration is likely.

## Conclusions

We simulated a positive landscape scale C sink in all our scenarios for the period 2000 to 2014 corroborating global (Pan et al. 2011) as well as Europe-wide (Luyssaert et al. 2010) assessments showing that temperate forests act as potent C sinks. However, a net C sink of the landscape is by no means guaranteed in the future. Among several factors, intensifications of forest disturbances (Seidl et al. 2014) might increase the frequency of periods with a net loss of C (Dobor et al. 2018). We showed that forest ground vegetation strongly compensated disturbance-induced C loss. At the landscape scale, however, tree regeneration was more important for NEP than ground vegetation. Capitalizing from this potential to its full extent will very likely be difficult considering the current degree of damage to tree regeneration by ungulate browsing in Austria (Reimoser and Reimoser 2010; Hangler 2017) and in other European temperate forest regions (Ammer 1996; Motta 1996; Firm et al. 2009; Reimoser and Reimoser 2010; Klopčič et al. 2017). In conclusion, our findings underline the importance of tree regeneration and ground vegetation in the context of the climate change mitigation potential of temperate forest ecosystems.

## Electronic supplementary material

Below is the link to the electronic supplementary material.

Supplementary material 1 (DOCX 502 kb)
