# Supplementary material for: Substantial understory contribution to the C sink of a European temperate mountain forest landscape
Source: Landsc Ecol. 2020 Feb 3;35(2):483–99. doi: 10.1007/s10980-019-00960-2 (PMC7045765; doi:10.1007/s10980-019-00960-2)
Supplement: Supplementary file 1 — Supplementary material 1 (DOCX 502 kb) [file 10980_2019_960_MOESM1_ESM.docx]

Supplementary Online Material for the manuscript

Dirnböck, T., Kraus, D., Grote, R., Klatt, S., Kobler, J., Schindlbacher, A., Seidl, R., Thom, D., Kiese, R. „Substantial understory contribution to the C sink of a European temperate mountain forest landscape”

S1. EcHy - Ecosystem Hydrology - An improved LandscapeDNDC hydrology model

S2. Details about LandscapeDNDC setup

S3. Observation data sets for model evaluation

# S1 EcHy - Ecosystem Hydrology - An improved LandscapeDNDC hydrology model

The official release of LandscapeDNDC in 2013 ([Haas et al., 2013](#_ENREF_1)), uses the hydrology model WatercycleDNDC that has been first described by Kiese et al. ([2011](#_ENREF_3)). WatercycleDNDC includes process-based descriptions of:

- Rain - Snowfall
- Interception
- Runoff
- Evapotranspiration
- Soil water movement

Vertical soil water movement within WatercycleDNDC is calculated by a so-called tipping bucket approach in which water in- and exfiltration for each discretized soil layer and simulated time step (1 hour in this study) are bound by air or water filled pore space, respectively. This restriction can lead to numerical problems under conditions of high precipitation in combination with low pore space. Under such conditions, WatercycleDNDC underestimates downward transport of water and thus overestimates water contents across the profile (see S1 Fig. 1). Due to very high stone contents and thus low pore space in some parts of the study region, an improved soil hydrology model, named EcHy, was developed. EcHy uses the WatercycleDNDC process descriptions for most water flux calculations (see above list) but replaces the numerical instable tipping bucket approach by an extended form of Darcy's law for multiphase flow:

$$q\left( z_{i} \right)= k_{f}(z_{i})\cdot k_{r}(z_{i})$$

The above equation assumes that gravity is the single driving force of water movement, i.e., neglecting capillary forces. The term $k_{r}$ stands for the relative permeability for which a van Genuchten parameterization is applied:

$$k_{r} = {wc}_{e} \left[ 1-\left( 1-{wc}_{e}^{\left( \frac{1}{m} \right)} \right)^{m} \right]^{2}$$

The terms ${wc}_{e}$ and $m$ are the effective water content and a soil texture dependent parameter, respectively. Both measures need to be provided as model input. The effective water content scales the actual water content between residual water content ${wc}_{r}$ and residual air-filled pore space $\phi_{r}$:

$${wc}_{e}=\frac{wc-{wc}_{r}}{\phi-\phi_{r}}$$

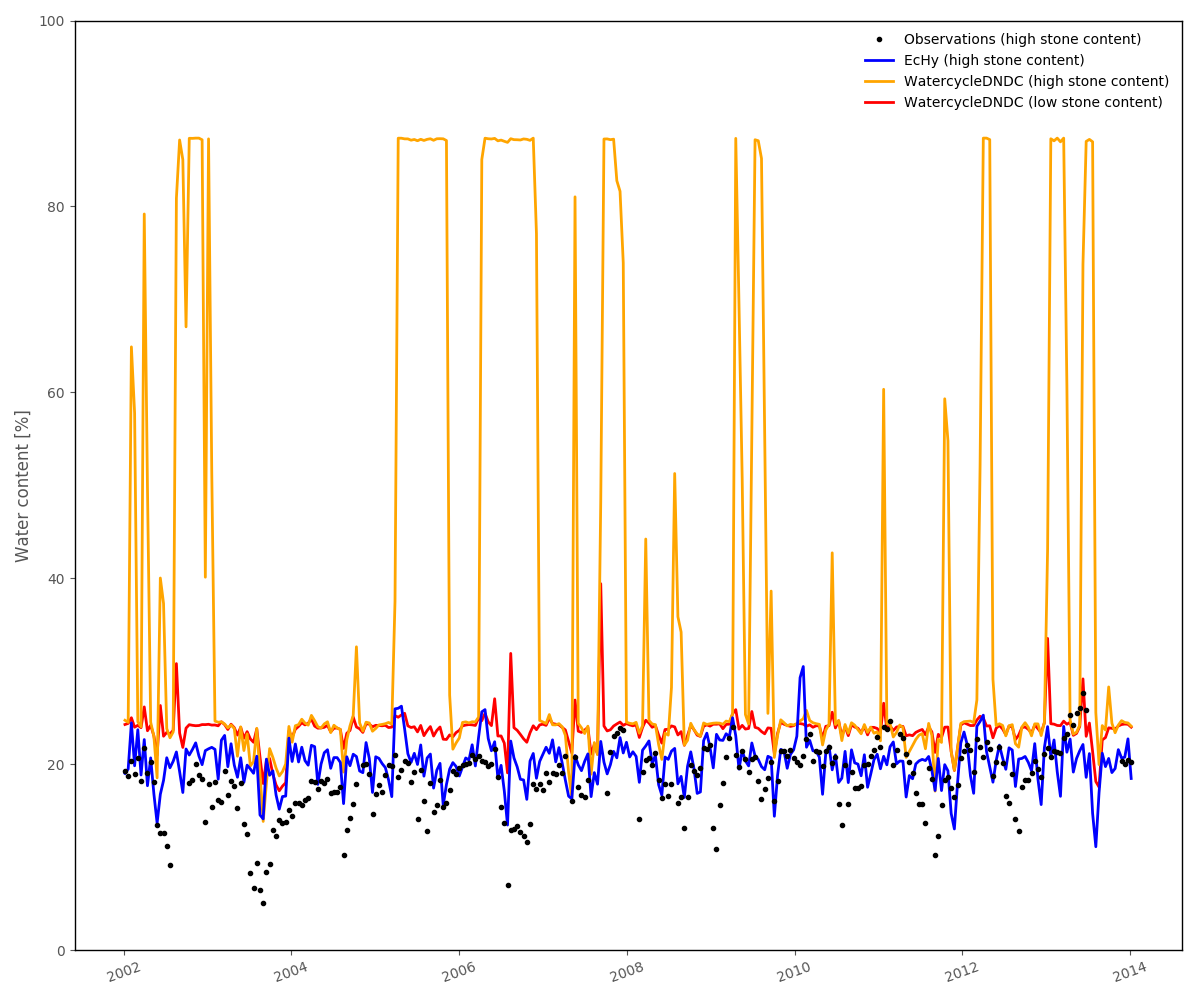
S1 Fig. 1: Simulated and observed soil water content in 5 cm soil depth at IP2. Simulations have been conducted with the soil hydrology models EcHy and WatercycleDNDC. For the WatercycleDNDC simulations, stone content has been decreased in order to illustrate the numerical problems related to increased stone contents.

# S2. Details about LandscapeDNDC setup

## Vegetation initialization

The tree data from Thom et al. ([2017](#_ENREF_9)) contained tree type- and age class-specific stand information, i.e., number of trees, diameter at breast height and height-diameter ratio. Respective information has been aggregated to the 100 x 100m simulation resolution by retaining total biomass but has been simplified regarding stand heterogeneity (see below). Excluding understory, the number of different tree species as well as the number of age classes for one tree species has been both limited by two, resulting in a maximum of four simulated cohorts. Depending on the scenario, two additional vegetation units, one for the forest ground vegetation and one for tree regeneration have been included. For tree species selection, the biomass was the single criterion, while for age class selection biomass as well as tree height was relevant. Scaling the number of individuals during simplification in order to match total original biomass ensured mass conservation. The following consecutive aggregation and simplification steps were applied:

1. Spatial additive aggregation from the original 10 x 10m resolution to the simulation resolution of 100 x 100m.
2. Replacing original tree species for which no suitable parameterization was available by suitable alternatives (S2 Table 1).
3. Estimation of total aboveground wood biomass per grid cell based on original stand structure, e.g., height and diameter, but updated tree species identity. Total aboveground wood biomass was estimated based on tree species-specific wood density DREF and the following equation for stem volume V (Zianis et al. 2005) where HTOP is the forest stand height (S2 Table 2):
4. Selection of two dominant species (depending on biomass) and age classes (depending on height).

S2 Table 1. Species replacements during vegetation initialization.

| Original species | Available | Replacement |
| --- | --- | --- |
| Abies alba | yes |  |
| Acer campestre |  | Acer pseudoplatanus |
| Acer platanoides |  | Acer pseudoplatanus |
| Acer pseudoplatanus | yes |  |
| Alnus glutinosa |  | Salix viminalis |
| Alnus incana |  | Salix viminalis |
| Alnus viridis |  | Salix viminalis |
| Betula pendula | yes |  |
| Carpinus betulus |  | Fagus sylvatica |
| Castanea sativa |  | Fagus sylvatica |
| Corylus avellana |  | Betula pendula |
| Fagus sylvatica | yes |  |
| Fraxinus excelsior | yes |  |
| Larix decidua | yes |  |
| Picea abies | yes |  |
| Pinus nigra |  | Pinus sylvestris |
| Populus nigra |  | Hybrid poplar |
| Populus tremula |  | Hybrid poplar |
| Pseudotsuga menzisii | yes |  |
| Quercus petraea |  | Quercus robur |
| Quercus pubescence | yes |  |
| Quercus robur | yes |  |
| Robinia pseudoacacia | yes |  |
| Salix caprea |  | Salix viminalis |
| Salix viminalis | yes |  |
| Sorbus aria |  | Betula pendula |
| Sorbus aucuparia |  | Betula pendula |
| Tilia cordata | yes |  |
| Tilia platyphyllos |  | Tilia cordata |
| Ulmus glabra |  | Fagus sylvatica |

*V =* DREF*^TAP1^ ** HTOP*^TAP2^ * e^TAP3^*

S2 Table 2. Tree species specific biomass parameters used for calculating stem volume

| Species | Wood  Density | TAP 1 | TAP 2 | TAP 3 |
| --- | --- | --- | --- | --- |
| Abies alba | 0.353 | 1.75 | 1.1 | -2.75 |
| Acer pseudoplatanus | 0.59 | 1.90 | 0.98 | -2.94 |
| Betula pendula | 0.43 | 1.89 | 0.27 | -1.07 |
| Fagus sylvatica | 0.68 | 1.41 (1.55) | 1.56 | -3.58 |
| Fraxinus excelsior | 0.64 | 1.95 | 0.77 | -2.48 |
| Larix decidua | 0.46 | 1.87 | 1.08 | -3.05 |
| Picea abies | 0.41 | 1.71 (1.55) | 1.11 | -2.76 |
| Pseudotsuga menzisii | 0.43 | 1.9 | 0.81 | -2.43 |
| Quercus pubescence | 0.57 | 1.95 | 0.75 | -2.4 |
| Quercus robur | 0.75 | 2.0 | 0.86 | -2.86 |
| Robinia pseudoacacia | 0.66 | 1.95 | 0.75 | -2.4 |
| Salix Viminalis | 0.5 | 1.95 | 0.75 | -2.4 |
| Tilia cordata | 0.42 | 1.95 | 0.75 | -2.4 |

Selection of dominant tree species and age classes was done according to the following procedure

1. For each grid cell, two dominant tree species were selected depending on biomass. All other tree species were neglected.
2. For each selected tree species, all age classes from the original stand structure were assigned to three defined height classes, i.e., 0m - 0.5 h_max_, 0.5 h_max_ - 0.8 h_max_, 0.8 h_max_ - h_max_, with h_max_ being the maximum height within one tree species. Within each of those three height classes an arbitrary number of age classes exists depending on the complexity of the original stand structure. From these age classes, only one dominant age class per height class was selected depending on biomass. As for now, each grid cell has been simplified to two dominant tree species having three dominant age classes each. From these three age classes only the two dominant age classes (depending on biomass) were finally chosen as cohorts for the simulation. In order to ensure mass conservation, the original number of individuals within each chosen age class was scaled matching total original biomass.

## Forest ground vegetation

In order to dynamically scale forest ground vegetation cover in relation to tree canopy dynamics in LandscapeDNDC we used vegetation data from 54 forest vegetation plots (10 x 10m) located in the 90 ha area of the LTER site Zöbelboden ([Helm, Essl, Mirtl, & Dirnböck, 2017](#_ENREF_2)) in the NPK. In each of these plots all vascular plant species (<0.6m height) were recorded in three years (between 1993 and 2014) together with their cover. Tree species were excluded in order to account only for small shrubs, herbs and grasses.

The forest ground vegetation cover A_h_ was then fitted to the overstory tree cover A_o_ (R²=0.35, p<0.001)

$$A_{h}=0.91 + 0.0016 A_{o} - 0.000067 A_{o}^{2}$$

where A_o_ was the sum of the canopy cover of the two dominant tree species bound by 100 %.

## Forest disturbance

The reconstruction of forest disturbance was based on the work of Thom et al. ([2017](#_ENREF_9)). First, they reconstructed the tree vegetation on the landscape in the year 1999, combining information from forest management plans (spatially available at the level of stand polygons, with a median stand size of 1.4 ha), sample-plot based forest inventory (on a 300 × 300m raster, 1122 sample plots in total), remotely sensed tree species distribution, as well as canopy height estimates from air-borne laser scanning (ALS, at 1m horizontal resolution). Inventory sample plots were imputed to stand polygons with corresponding species distribution and canopy height. Subsequently, forest structure was derived from sample plots, and tree positions within stands determined based on ALS data. In total, more than 2106 trees from 17 different species were initialized on the 13865 ha of stockable forest area. Subsequently, tree species development from 1999 onwards was simulated with iLand ([Seidl, Rammer, Scheller, & Spies, 2012](#_ENREF_8)). Disturbances during that period were simulated using disturbance records from the managing agency of the Kalkalpen National Park service as calibration data. For simplification, disturbances (corresponding to timber volume killed) have been assigned to single fixed days of year in LandscapeDNDC, i.e., 15^th^ January for snow disturbance, 1^st^ September for windfall and 15^th^ September for bark beetle attack.

## Soil initialization

Soil initialization was derived from an existing inventory of 137 soil investigations spread over the complete National Park. Out of this, 48 soil investigations were discarded due to insufficient information. Each accepted soil investigation was classified depending on soil type, soil depth, elevation, aspect and slope (S2 Table 3), which has been available area-wide from (Kobler 2004).

S2. Table 3. Classification rules for soil initialization

| Classification | Class I | Class II | Class III |
| --- | --- | --- | --- |
| Soil type | Leptosols | Cambisols | Stagnosols |
| Soil depth | <=30cm | >30cm |  |
| Elevation | ≤ 700m | >700m and ≤ 1400m | > 1400m |
| Aspect | flat | NW->N->E | SE -> S -> W |
| Slope | ≤ 20% | > 20% and ≤ 60% | > 60% |

From all soil investigations, which fulfil the classification rules for a given simulated grid cell, the one having the median soil organic carbon has been finally chosen as most representative. In a subsequent processing step, the soil depth of the original soil investigation has been adjusted in order to match the soil depth as given area-wide by (Kobler 2004). Since only a limited number of soil investigations recorded bulk density (n=60), missing values were gap filled by an exponential regression between soil organic carbon SOC and bulk density BD (S2 Figure 1):

$$BD=1.275 e^{-0.0686 SOC}$$

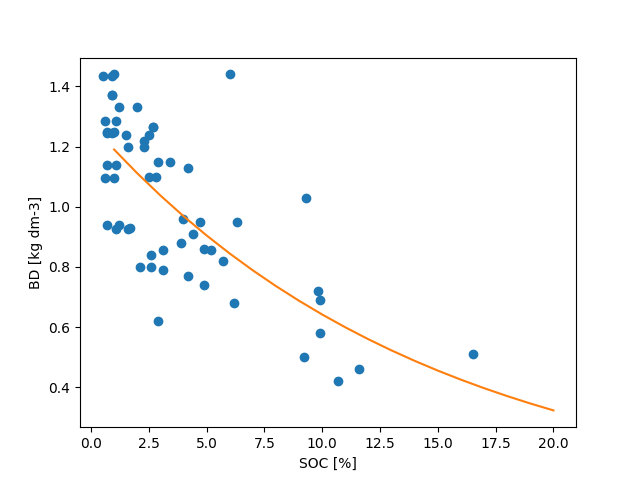


S2 Figure 1. Exponential regression between soil organic carbon SOC and bulk density BD (R^2^ = 0.58)

# S3 Observation data sets for model evaluation

Tree growth at IP1 (1996 – 2009) and IP2 (1997 – 2015) was calculated based upon weekly stem diameter measurements (IP1: spruce n=13, beech n=5; IP2: beech n=12, spruce n=6; Dial Dendro Relaskop-Technik GmbH) which were scaled up to the entire stand using tree inventories from the years 2003, 2007, and 2014. During inventories, tree height and diameter at breast height were measured. Missing tree heights and tree heights for every weekly dendrometer measurement were calculated from stem diameter using linear regression. Individual tree loss due to windthrow and bark beetle was recorded and subtracted from the total forest stand biomass. Stem wood volume (fresh mass) was estimated based on tree species-specific volume equations ([Pollanschütz, 1974](#_ENREF_6)). The resulting volume was lowered by a shrinking factor to obtain the oven-dry volume (spruce: 0.118, beech: 0.179). Subsequently, conversion factors (spruce: 0.41 t m^-3^, beech: 0.68 t m^-3^) were used to convert the corrected volumes to stem wood biomass.

Soil water content (SWC) was averaged to daily values from half-hourly data (measured since 2001) of time domain reflectometry (TDR) sensors (IP1: 2 x 5 cm and 2 x 20 cm depth; IP2: 5 x 5 cm depth).

Soil respiration (sum of autotrophic R_a_ and heterotrophic respiration R_h_) was measured along transects consisting of 8 (IP1) and 9 (IP2) individual plots (spacing ~ 5m), each with 2-3 randomly distributed collars (10 cm diameter, 4 cm height). Soil CO_2_ efflux was measured with an EGM-4 infrared gas analyzer connected to a SRC-1 soil respiration chamber (PP-Systems, Amesbury, USA), first averaged for each of the plots and then aggregated to the IP1 (late 2009 to late 2011, see Kobler et al. ([2015](#_ENREF_4))) and IP2 (early 2015 to late 2016).

For a stratified subsample of 9 of the 54 vegetation records (see S2), the entire herb and grass layers was clipped and weighed in order to determine understory biomass B_h_ ([Johannes Kobler et al., 2019](#_ENREF_5)). A log linear model was then fitted (clipped plots) with understory biomass as the response variable, and visually estimated percent plant cover A_h_ and mean vegetation height (weighed by single species cover) as the predictor variables (R²=0.65, p=0.027). Species specific vegetation height was taken from trait data ([Seebacher, Dirnböck, Dullinger, & Karrer, 2012](#_ENREF_7)). Originally, we did also use the mean specific leaf area (SLA) of all species as an additional predictor but its inclusion did not result in a significantly better model fit. This model was applied to vegetation records in IP1 and IP2 as well as the 54 10 x 10 m vegetation records, representing the summer seasonal biomass estimates. At IP1 three records including overstory disturbances in between could be compared with model results, at IP2, only one year (a second record in 2007 was not representative for the modelled plot) (see Table 2 in the manuscript). In addition, we used the estimated biomass accumulations 1 to 20 years after disturbance (tree cover loss >30 and >50%) among the 54 records. When simulating similar overstory disturbances in IP1 and IP2, understory biomass dynamics modelled with LandscapeDNDC could be parameterized (see Table 3 in the manuscript).

S3 Figure 1. Simulated and measured data of the two intensively studied sites IP1 (A, B) and IP2 (C, D). Modelled stem dry weight biomass (A, C) is shown per tree species (shades), measurements are given for beech and spruce (solid lines). Modelled total and heterotrophic soil respiration is shown as solid lines, measured total soil respiration (B, D) is shown as mean values (±SE).

# References

Haas, E., Klatt, S., Fröhlich, A., Kraft, P., Werner, C., Kiese, R., . . . Butterbach-Bahl, K. (2013). LandscapeDNDC: a process model for simulation of biosphere–atmosphere–hydrosphere exchange processes at site and regional scale. *Landscape Ecology, 28*(4), 615-636. doi:10.1007/s10980-012-9772-x

Helm, N., Essl, F., Mirtl, M., & Dirnböck, T. (2017). Multiple environmental changes drive forest floor vegetation in a temperate mountain forest. *Ecology and Evolution, 7*(7), 2155-2168. doi:10.1002/ece3.2801

Kiese, R., Heinzeller, C., Werner, C., Wochele, S., Grote, R., & Butterbach-Bahl, K. (2011). Quantification of nitrate leaching from German forest ecosystems by use of a process oriented biogeochemical model. *Environmental Pollution, 159*(11), 3204-3214. doi:10.1016/j.envpol.2011.05.004

Kobler, J., Jandl, R., Dirnböck, T., Mirtl, M., & Schindlbacher, A. (2015). Effects of stand patchiness due to windthrow and bark beetle abatement measures on soil CO2 efflux and net ecosystem productivity of a managed temperate mountain forest. *European Journal of Forest Research*, 1-10. doi:10.1007/s10342-015-0882-2

Kobler, J., Zehetgruber, B., Dirnböck, T., Jandl, R., Mirtl, M., & Schindlbacher, A. (2019). Effects of aspect and altitude on carbon cycling processes in a temperate mountain forest catchment. *Landscape Ecology, accepted*.

Pollanschütz, J. (1974). Formzahlfunktionen der Hauptbaumarten Österreichs. *Informationsdienst Forstliche Bundesversuchsanstalt, 153*, 341-343.

Seebacher, D., Dirnböck, T., Dullinger, S., & Karrer, G. (2012). Small-scale variation of plant traits in a temperate forest understorey in relation to environmental conditions and disturbance. *Stapfia, 97*, 153-168.

Seidl, R., Rammer, W., Scheller, R. M., & Spies, T. A. (2012). An individual-based process model to simulate landscape-scale forest ecosystem dynamics. *Ecological Modelling, 231*(0), 87-100. doi:<http://dx.doi.org/10.1016/j.ecolmodel.2012.02.015>

Thom, D., Rammer, W., & Seidl, R. (2017). The impact of future forest dynamics on climate: interactive effects of changing vegetation and disturbance regimes. *Ecological Monographs, 87*(4), 665-684. doi:10.1002/ecm.1272
